# Air quality and mental illness: role of bioaerosols, causal mechanisms and research priorities

**DOI:** 10.1192/bjo.2024.724

**Published:** 2024-09-19

**Authors:** Kamaldeep Bhui, Marcella Ucci, Prashant Kumar, Simon K. Jackson, Corinne Whitby, Ian Colbeck, Christian Pfrang, Zaheer A. Nasir, Frederic Coulon

**Affiliations:** Department of Psychiatry and Nuffield Department of Primary Care Health Science, Wadham College, University of Oxford, Oxford, UK; and Global Policy Institute, Queen Mary University of London, London, UK; UCL Institute for Environmental Design and Engineering, London, UK; Global Centre for Clean Air Research, School of Sustainability, Civil and Environmental Engineering, Faculty of Engineering and Physical Sciences, University of Surrey, Guildford, UK; School of Biomedical Sciences, Faculty of Health, University of Plymouth, Plymouth, UK; School of Life Sciences, University of Essex, Colchester, UK; School of Geography, Earth and Environmental Sciences, University of Birmingham, Birmingham, UK; School of Water, Energy and Environment, Cranfield University, Cranfield, UK

**Keywords:** Bioaerosols, exposure, health impacts, air pollution, indoor and outdoor air

## Abstract

**Background:**

Poor air quality can both trigger and aggravate lung and heart conditions, as well as affecting child development. It can even lead to neurological and mental health problems. However, the precise mechanisms by which air pollution affect human health are not well understood.

**Aims:**

To promote interdisciplinary dialogue and better research based on a critical summary of evidence on air quality and health, with an emphasis on mental health, and to do so with a special focus on bioaerosols as a common but neglected air constituent.

**Method:**

A rapid narrative review and interdisciplinary expert consultation, as is recommended for a complex and rapidly changing field of research.

**Results:**

The research methods used to assess exposures and outcomes vary across different fields of study, resulting in a disconnect in bioaerosol and health research. We make recommendations to enhance the evidence base by standardising measures of exposure to both particulate matter in general and bioaerosols specifically. We present methods for assessing mental health and ideal designs. There is less research on bioaerosols, and we provide specific ways of measuring exposure to these. We suggest research designs for investigating causal mechanisms as important intermediate steps before undertaking larger-scale and definitive studies.

**Conclusions:**

We propose methods for exposure and outcome measurement, as well as optimal research designs to inform the development of standards for undertaking and reporting research and for future policy.

Poor air quality is a risk factor for a range of non-communicable diseases, including mental health conditions.^1^ Although much of the evidence is based on ecological data,^2,3^ there are also noteworthy intriguing service use and incidence studies suggesting important connections between air pollution and adverse mental health outcomes.^4,5^ For instance, poor housing conditions and fungal exposure can directly and indirectly affect mental health through respiratory issues.^6,7^ Despite limitations such as diverse outcomes and exposure assessment methods, several studies emphasise critical public health implications that we overlook at our peril, particularly amid the global climate crisis exacerbating air quality and health concerns.^8^ It is crucial for preventive actions to rely on the best available evidence rather than desired evidence. However, policy makers and legislators often face the challenge of basing decisions on known facts rather than providing more certainty when persuading stakeholders to act. Therefore, prioritising better research over sheer volume of research is imperative. Acknowledging these realities, our collective work synthesises insights from experts across diverse disciplines working closely with other global research groups. This interdisciplinary review aims to examine the relationship between poor air quality and non-communicable diseases, specifically focusing on mental health conditions and the underexplored health implications of bioaerosols (i.e. suspensions of airborne particulate matter of biological origin (BioPM)). Bioaerosols or BioPM include microorganisms (bacteria, fungi/mould, viruses) and their products (e.g. endotoxins, cell fragments and microbial volatile organic compounds).

The composition of the air we breathe, both indoors and outdoors, comprises various gaseous (NO, NO_2_, SO_2_, CO, O_3,_) and particulate pollutants, among which particulate matter stands out as a significant public health concern. Particulate matter emissions can result either directly from natural sources (e.g. volcanoes, sea spray, wildfires) or human activities (household combustion, transport, industrial facilities, agricultural activities) or through secondary formation in the atmosphere by chemical reactions. These emissions vary in size and composition, encompassing ultrafine (<100 nm), fine (less than 2.5 μm in aerodynamic diameter: PM_2.5_) and coarse (less than 10 μm in diameter: PM_10_) particles, as well as BioPM.

The relationship between mental health and air pollution is growing.^9–11^ Air pollution contributes to 9 million deaths a year worldwide.^12^ It has lifelong effects on vulnerable people such as older people, pregnant women and children, and on those with existing medical conditions, who are disproportionately affected by air pollution. Particulate matter is of particular concern, especially PM_2.5_.^13^ Exposure to excessive PM_2.5_, even in the short term, can cause breathing difficulties, especially in individuals with pre-existing lung conditions such as asthma or chronic obstructive pulmonary disease.^14,15^ The prevalence of asthma and allergies has increased in recent decades, particularly in urbanised areas.^16^ Research has shown that air pollution may interact with airborne allergens, enhancing the risk of atopic sensitisation and exacerbation of symptoms.^17,18^ Although these studies are on respiratory conditions and not direct effects on mental state, people with multimorbidities including respiratory and mental conditions are likely to be affected; for example, people with psychosis are already more likely to have poor lung function.^19^ People with asthma who are taking antipsychotics are at greater risk of mortality compared with those not taking these medications.^20^ Therefore, there are plausible pathways to poor health outcomes for those with severe mental illness, impaired lung function and/or respiratory disease, if they encounter high levels of air pollution. Indeed, there are direct postulated pathways between air pollution and mental illness through inflammatory responses in the brain, as well as indirect pathways including systemic inflammation leading to brain inflammation, long term medical conditions leading to poor mental health, and *vice versa* (Fig. 1).

**Fig. 1:**
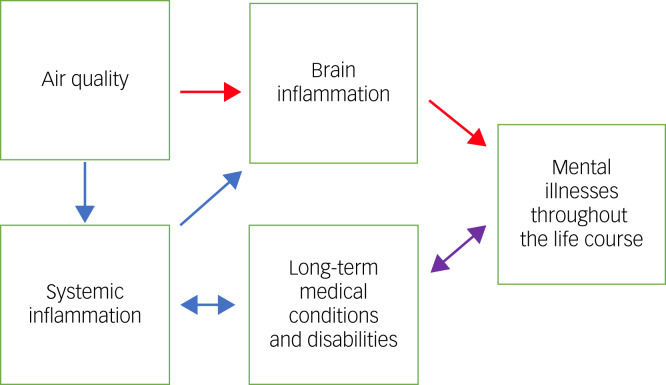
Air quality and pathways to mental illnesses.

In addition to particulate matter, bioaerosols bring additional challenges in terms of health risk. Bioaerosols are the biological fraction of particulate matter and are a complex mixture of bacteria, viruses and fungi, or parts of living organisms, such as pollen, spores, endotoxins from bacterial cells and mycotoxins from fungi. Of particular concern is the small size and mass of BioPM, which means they are easily transported over distances facilitating the rapid spread of microorganisms and their genetic material. BioPM are ubiquitous in indoor and outdoor environments and are also an important transmission route for infectious and sensitisation agents, yet knowledge of their role in human health is currently limited.

Our work is necessarily a rapid narrative review rather than a systematic review, undertaken with a network of experts to gather evidence across disparate fields and bodies of knowledge; this approach is appropriate when there is a rapidly changing and complex literature that is not easily identified and on which there will be disagreement.^1,21,22^

## BioPM and health

BioPM contribute to 16.5% of PM_2.5_ and 16.3% of PM_10_.^23^ Although there have been advances in knowledge with regard to the physical properties (mass, number, volume) and chemical composition (chemical speciation) of particulate matter, progress relating to the characterisation of BioPM and the interactions between their abiotic and biological components has been limited. This significantly limits our understanding of the mechanisms of toxicity and the impact of particulate matter in general – and BioPM specifically – on public health.

Pandemic outbreaks of influenza and SARS CoV-2, as well as bio-terror attacks, have raised the priority of research on BioPM, about which less is known. Urban environments are characterised by multiple sources of air pollutants, including BioPM, that influence indoor and outdoor air quality. Indoor air quality is a critical factor in estimates of total exposure to air pollutants, but it is still less emphasised in policy and practice.^7^ Indoor environments expose humans to sources such as buildings and household materials. Large numbers of microbes live naturally on our skin and other body surfaces. Some of these are released into the air spontaneously through air movements or through coughing, sneezing, talking or breathing. In poorly ventilated enclosed spaces, there is a greater potential of airborne disease transmission. Harmful air pollutant emissions arise from cooking activities, domestic cleaning products, household heating, dampness alongside fungi, bacteria, viruses and other BioPM.^24,25^ COVID-19 is transmitted through air and so might be considered to be a bioaerosol. Mortality rates among people with psychosis have been reported to be higher among those exposed to COVID-19, implicating respiratory and other comorbid conditions.^26,27^ However, the majority of exposures to BioPM are benign, and loss of exposure to key microbes can also be associated with problems of the gut, skin and mental health.^28^ That is, some BioPM may even promote health; for example, diverse aerobiome characteristics are associated with healthier immune responses, fewer allergic reactions, and even reduced blood pressure and enhanced natural killer cell activity.^29^

## Knowledge gaps

BioAirNet is a network funded by UK Research & Innovation (UKRI)/Natural Environment Research Council (NERC) specifically investigating BioPM in relation to human health, while assessing the evidence base on air quality and effects on health more generally. Understanding the impact of BioPM requires those already steeped in particulate matter research and practice to extend their paradigm to consider some of the unique challenges presented by BioPM. Through interdisciplinary dialogues, expert consensus meetings and rapid evidence syntheses, BioAirNet has identified key priorities, including understanding causality and causal mechanisms, enhancing exposure measurement techniques, and developing innovative research methods for assessing the health effects of air pollution.

Regarding interdisciplinary perspectives on causal inferences, there is a lack of consensus on appropriate standards of measurement of particulate matter and mental health, and on the ways in which causality might be assessed.^30–32^ Indeed, causal inferences in epidemiology rely on formal criteria, such as those of Bradford Hill: strength of association, consistency, specificity, temporality, biological gradient, plausibility, coherence, experiment and analogy.^33^ However, these were conceived when molecular science was not as advanced, and new ways of interpreting causality are now required, including paying attention to molecular mechanisms where, for example, judgements on strength of association must be made in the context of the levels of probability as well as advances in analytic tools and computing power.^34^ Yet, we also need to show some humility when encountering complex layered systems, which are mutually constitutive, involving thousands of chemical interactions and substances which affect mental states; these are also likely to be influenced by environmental and neuroscientific affordances, including looping effects connecting distinct system elements.^35^ Indeed, Fedak et al^34^ argue that ‘the criteria should not be used as a heuristic for assessing causation in a vacuum; rather they should be viewed as a list of possible considerations meant to generate thoughtful discourse among researchers from diverse scientific fields’.

Therefore, in this review, we acknowledge caution surrounding discussions of causality while also asserting that there exists sufficient evidence for the practice and policy community to be aware of and develop robust future plans in response to this emerging evidence. An additional challenge is that what is assumed knowledge in one discipline may be contested or novel in another – or even disbelieved, as it brings in knowledge from vastly differing notions of evidence and causality. Indeed, there are even disputes about the causes of depression and which neurotransmitters are accountable, revealing differing frames for judging causality in ultra-complex of human–environmental systems.^36^

Identification of critical and plausible mechanisms by which particulate matter, particularly BioPM, might lead to poor health can support causal theories and prevention efforts. These mechanisms may be biological, social, psychological and also geographical, and interactions of all of these. More studies might use animal or laboratory models to investigate biological and chemical processes implicated in potential mechanisms.

### Critical mechanisms

PM_2.5_ can enter the lungs and bloodstream, reaching brain tissue to produce immune and inflammatory responses locally^37^, and may lead to cellular damage, depending on size and chemical or biological properties. We focus here on inflammation and immune mechanisms, as these are increasingly linked to poor mental and physical health; indeed, reverse causality between mental and physical health and shared aetiological mechanisms suggest we should look at the two together and not adopt the Cartesian dichotomy which has bedevilled health research. People with pre-existing mental illnesses are vulnerable and already have a life expectancy shortened by 15–20 years owing to long-term physical illnesses.^38^

Endotoxins are pro-inflammatory, and metabolites from BioPM may be toxic. Mechanisms include oxidative stress and immune dysregulation, for example, by release of cytokines, with an impact on T and B cells that form part of the immune response, and disruption in the balance of immunoglobulins that fight infection and invasion by contaminating substances. There may be direct carcinogenic risks or toxicity to cells, from lead and cadmium, for example.^9^ These processes can harm brain function and also lead to depression and cognitive decline. Particulate matter may damage respiratory cells, provoking a local immune response, and changes in the lung microbiome, in turn, can affect the gut microbiome.^39^ This can worsen existing conditions or precipitate new episodes of physical and mental illness.

Adversity and poverty can produce inflammation in mothers, the fetus *in utero*, young children and young adults, as well throughout the life course.^40^ Inflammation can lead to depressive experiences, mental illnesses and physical illnesses. Depression, both as a clinical diagnosis and as less severe symptoms, includes pessimism, poor self-care and a lack of motivation and can thus compound the adverse effects of poverty by reducing social support and risking unemployment. Indeed, poverty and food scarcity are known to directly influence cognitive decisions,^41^ and this makes brain health vulnerable to additional risks such as poor air quality.

There are direct effects of particulate matter and/or BioPM and inflammation on health; however, epigenetic mechanisms can also be activated by adversity to alter immune responses, leading to poor mental and physical health. For example, early life adversity, trauma and poverty can lead to premature ageing and a greater risk of multiple long-term conditions.^42–45^ Studies show that even exposure to particulate matter in the prenatal and perinatal period can affect later health.

These webs of causation and social determinants of poor health are more common in urban environments.^46^ Among deprived inner-city areas, health risk behaviours are more common and can add additional harms, for example, through smoking and excessive use of alcohol, a lack of physical activity, greater risk of adverse childhood experiences, poor early-life care, neglect, parental mental illness, poor educational achievement and school exclusion, and incarceration.^47^ These all promote ‘inflammogenic’ environments that are vulnerabilities for additional risk exposures. Indeed, these are the mechanisms by which COVID-19 was proposed to more specifically affect people facing multiple social inequalities and marginalisation.^48^ Therefore, among people with established physical or mental illnesses, particulate matter can exacerbate and precipitate additional episodes of ill health requiring more specialist and intensive interventions.^49^

### Quantification of BioPM exposures

**Fig. 2:**
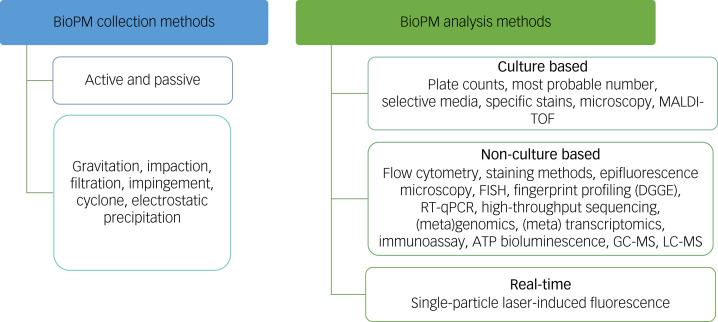
Overview of BioPM (particulate matter of biological origin) collection and analysis methods (adapted from ref. ^50^). MALDI-TOF, matrix-assisted laser desorption/ionisation; FISH, fluorescence *in situ* hybridisation; DGGE, denaturing gradient gel electrophoresis; RT-qPCR, real-time quantitative polymerase chain reaction; ATP, adenosine triphosphate; GC-MS, gas chromatography-mass spectrometry; LC-MS, liquid chromatography-mass spectrometry.

The existing evidence base on BioPM stems from disconnected scientific disciplines and sectoral foci, each with their own perspectives and methods. Currently, detection and characterisation of BioPM is based largely on culture-based microbiology, microscopy (spores, pollen), bioassays (endotoxin, 1,3-β-d-glucan), chromatography and mass spectroscopy for specific biochemicals (ergosterol and mannitol/arbitol, 3-hydroxy fatty acids, microbial volatile organic compounds, phospholipid-derived fatty acids, and molecular microbiology (DNA, RNA). Each sampling and analysis method has various advantages and disadvantages, which have been thoroughly discussed previously.^50^ Moreover, there are currently no standardised protocols for bioaerosol sampling and analysis, making it difficult to compare studies and assess dose–effect relationships.^50^ To better understand the impact of bioaerosol exposure on human health, comprehensive collection and analysis methods are needed to detect, characterise and quantify BioPM and its interactions with other pollutants.^50^ The most frequently used collection methods include gravitation, impaction, impingement, cyclone and filtration, allowing a range of analysis options. Methods to characterise BioPM in real time are also emerging (Fig. 2).^50^ In general, the most appropriate method must obtain a representative sample of the environment being investigated and will depend upon the taxa of interest, preservation of sample integrity and the purpose of the study.^50^ To achieve this, bespoke combinations of methods are needed that are tailored to the environment, context, downstream analysis and research questions under investigation.^50^

Each BioPM collection and analysis method has advantages and disadvantages. Although significant progress in bioaerosol collection and analysis has been achieved over the past two decades, there is still no consensus on collection or any standardised analysis method for a particular context or environment. This makes it difficult for researchers to compare data across studies and for regulators to set meaningful exposure limits for BioPM for a particular environment. Although some headway has been made towards addressing this issue,^51^ more research is needed in this area. Analytical methods to provide real-time detection of BioPM need further development to improve their capabilities for rapid identification of BioPM.

### Innovation in studies of mental health impacts of air pollution

We should improve measurement and evidence of harms for different exposures to define thresholds by which one might define ‘clean’ or ‘healthy’ air. Even this terminology attracts ethical and philosophical questions about what the public might understand and how we apply criteria to define ‘clean’ or ‘healthy’. We need to develop ways of reliably measuring air particles, including bioaerosols, with sufficient precision to build evidence on what is healthy. Given the lack of knowledge on BioPM and the widespread exposure, we need better methods of measurement. More specifically, we need to establish a consensus on BioPM sampling and analysis methods. This includes quantification and characterisation of BioPM in indoor and outdoor locations.^52,53^ Separate studies might consider measurement of exposures in the most vulnerable groups, for example, those living in areas of high exposure near to farmland or waste disposal,^52^ and where populations already carry a higher burden of health problems.

In our introduction and our previous paper, we set out the evidence on how air pollution in general might drive poor health, including the onset of new mental illnesses and exacerbation of existing mental illnesses.^1^ These studies may be epidemiological, ecological or observational clinical studies and trials of interventions (medical, social or policy). Ecological studies are weaker owing to high residual confounding; however, they are an important step towards identifying mechanisms of action and prevention. Thus, epidemiological studies in populations and new cohorts collected specifically for testing the effects of air pollution are the most likely to yield valid data in which individual and area confounders can be considered and immunological mechanisms can also be investigated. Studies of clinical populations with poor mental health and chronic medical conditions could reveal how air pollution affects ongoing health and might lead to relapse and greater service use.

Mental illnesses can be measured using several valid methods, and this consists of measuring distinct diagnosed conditions (including psychoses, depression, anxiety and personality difficulties, as well as degenerative brain conditions such as dementia or cognitive impairment associated with neurological conditions, e.g. epilepsy, Parkinson's disease). Studies assessing associations with mental illness will need to better define which types of mental illness are considered. Psychoses are rarer, and neurological conditions specifically carry additional risks for poor mental health, and there may be shared mechanisms and pathway to several mental illnesses.^54^

Many non-specialists are unaware of how to assess mental illnesses. Although some biomarker research is underway, there are not yet any biomarkers that have sufficient specificity and sensitivity for diagnosis. Most diagnoses are based on assessment of experiences and symptoms using the following methods.

- Self-report screening and outcome measures, for example, Generalized Anxiety Disorder 7 to measure anxiety and nine-item Patient Health Questionnaire to measure depression.^55^
- Interviews using structured questionnaires that generate valid ICD-10 diagnoses, for example; the clinical interview schedule is one such measure.^56^
- Primary care and health service records including verbatim accounts can be mined using artificial intelligence software, but there is variation in data quality.^57,58^
- Social care and criminal justice systems have their own administrative data systems, and these can be linked with other data systems.^59^
- Prescription of psychotropic medication is similarly noted in several national data-sets, as well as local cohorts.^60^

Pathways into care and help-seeking are known to vary by age, gender and ethnicity.^61^ In terms of estimating levels of mental health problems, there are discrepancies between hospital and primary care and population levels of poor mental health, given that not everyone seeks help from primary care. Even fewer people seek help from or are referred to hospital care; rather, most will seek help in the community or from primary care.^62^ Indeed, many people with poor mental health may not be recognised as having poor mental health, especially in low-income countries.^63^ Therefore, population research is necessary to provide a true picture of levels of mental illness and the impact of poor air quality on mental health. We need better methods to engage underrepresented groups in research, for example, ethnic minorities and those living in poorer areas, the very people who are likely to be most affected.

Two areas of technology development are likely to have a major impact on exposure and outcome measurement, wearable technology and artificial intelligence as a research tool.^64^ Recent advances in technologies make it possible to wear devices that measure respiration, heart rates and blood oxygen and even take a basic electrocardiogram; some apps can pose questions about mental health on a hour-by-hour or daily basis. These ‘wearable’ and ‘experience sampling’ approaches permit real-time collection of data on emotional states, behaviours, eating patterns (including use of alcohol, medication and smoking) and physiological measurements that might indicate anxiety and distress.^65^ Experience sampling can also be used to record self-reported hallucinations, depressive thinking or paranoid beliefs.

Wearables offer advantages in that real-time and spatial data can be gathered that provide a better approximation of real-world exposures to air particles, as well as mental health and physiological parameters. For example, personal exposure to air pollution can be measured, and people can rate their mood or levels of anxiety. Wearables also offer opportunities for health-protective and health-promoting actions, for example, avoiding high-pollution areas if there are pre-existing conditions. For example, a person with asthma could choose to do sport, for example, only when pollution levels are not high. Artificial intelligence methods might also provide methods for exposure measurement and prediction.^66^ Such techniques have been used to predict hospital admission for cardiorespiratory conditions^67^ and are being used to better refine classification of mental illnesses,^68^ with aspirations to generate better evidence on the links between air pollution and mental illness.^69^

## Conclusions

To foster effective research and collaboration across different disciplines, there is a pressing need for a shared strategy including shared priorities and established standards for measuring and reporting exposure and outcomes. Although we have set out our conclusions (above), this requires a global and regional effort across different scientific areas of interest, from genetics and molecular studies to research in animal models, population and clinical studies, and environmental sciences. Currently, the greatest obstacle to progress is a scarcity of widely reliable methods that are practical and have been studied in each research niche, yet permit information to flow from genetic and/or molecular findings through to environmental research endeavours and *vice versa*. For example, for human studies we propose studies of clinical populations with pre-existing conditions, as well as studies of aetiology and incidence in high-exposure areas compared with low-exposure areas, allowing for quasi-experimental intervention studies and naturalistic investigations. These could be on indoor and outdoor, open or closed spaces, institutions, and small areas, as well as closer investigations during epidemics around the world. Infectious disease paradigms have heightened interest in bioaerosols, and the climate crisis is clearly raising air pollution as a priority alongside natural disasters and the consequences for human health of geopolitical disruption. The limitations we outline underscore the importance of interdisciplinary analysis to evaluate the plausibility and significance of specific mechanisms for interventions in healthcare, as well as for informing preventive public health policies and practices. As part of the work of BioAirNet, we are working with other research groups in the UK, Canada and France, and we welcome further collaborations to build consensus around research agendas, methods, and standardised common measures and reporting standards.

## Data Availability

Data availability is not applicable to this article as no new data were created or analysed in this study.
